# Molecular Analysis of AFP and HSA Interactions with PTEN Protein

**DOI:** 10.1155/2015/256916

**Published:** 2015-05-20

**Authors:** Mingyue Zhu, Bo Lin, Peng Zhou, Mengsen Li

**Affiliations:** ^1^College of Agriculture, Hainan University, Haikou, Hainan 570228, China; ^2^Hainan Provincial Key Laboratory of Carcinogenesis and Intervention, Hainan Medical College, Haikou, Hainan 571199, China; ^3^Key Laboratory of Molecular Biology, Hainan Medical College, Haikou, Hainan 571199, China; ^4^Institution of Tumor, Hainan Medical College, Haikou, Hainan 570102, China

## Abstract

Human cytoplasmic alpha-fetoprotein (AFP) has been classified as a member of the albuminoid gene family. The protein sequence of AFP has significant homology to that of human serum albumin (HSA), but its biological characteristics are vastly different from HSA. The AFP functions as a regulator in the phosphatidylinositol 3-kinase (PI3K)/protein kinase B (AKT) pathway, but HSA plays a key role as a transport protein. To probe their molecular mechanisms, we have applied colocalization, coimmunoprecipitation (co-IP), and molecular docking approaches to analyze the differences between AFP and HSA. The data from colocalization and co-IP displayed a strong interaction between AFP and PTEN (phosphatase and tensin homolog), demonstrating that AFP did bind to PTEN, but HSA did not. The molecular docking study further showed that the AFP domains I and III could contact with PTEN. *In silicon* substitutions of AFP binding site residues at position 490M/K and 105L/R corresponding to residues K490 and R105 in HSA resulted in steric clashes with PTEN residues R150 and K46, respectively. These steric clashes may explain the reason why HSA cannot bind to PTEN. Ultimately, the experimental results and the molecular modeling data from the interactions of AFP and HSA with PTEN will help us to identify targets for designing drugs and vaccines against human hepatocellular carcinoma.

## 1. Introduction

Hepatocellular carcinoma (HCC) is a major human cancer disease that threatens human life and health; HCC causes cancer-related death worldwide with approximately 600,000 people annually [1, 2]. The human alpha-fetoprotein (AFP) was assumed to be the early serum growth factor in the fetal liver development, but is also frequently found hepatic oncogenesis, such as in HCC patients. Thus, the AFP is considered to be a diagnostic marker for HCC. However, the actual biological roles played by this cytoplasmic AFP remain elusive [3].

AFP is known as an oncofetal protein that is highly expressed during embryonic and fetal development, but its levels are drastically reduced in the prenatal period. AFP has a molecular weight of 67 kDa and is a single peptide chain of 609 residues with 3–5% carbohydrate-glycan content. AFP amino acid sequence is similar to albumin, gc-globulin, or alpha-albumin. AFP has been classified as a member of the albuminoid gene family [4–7]. Human serum albumin (HSA) is the most abundant protein in the blood and plays a key role in the transport of fatty acids, metabolites, and drugs [8, 9], but AFP is known to serve as a kind of growth regulator [10–12]. It is interesting to know that, while these two protein sequences are similar, their functions are very different.

In the last decade, a great progress has been made in the study of AFP functions and roles. In addition to its application in the HCC diagnosis, AFP has been defined as a type of growth regulator during oncogenic growth and tumor progression [13]. We have recently provided some evidence to illustrate that cytoplasmic AFP may function in the upregulation of PI3K/AFT pathways in human hepatocellular carcinoma cells through binding to PTEN (phosphatase and tensin homolog) [14]. This function makes AFP different from albumin and other proteins.

PTEN is a tumor suppressor which can antagonize phosphatidylinositol-4,5-bisphosphate 3-kinase (PI3K) activity. PTEN can hydrolyze 3-phosphate on phosphatidylinositol-3,4,5,-triphosphate (PIP3). PI (3,4,5) P3 is the second lipid messenger produced by the phosphoinositide 3-kinase (PI3K) and activates downstream effectors. These include the Akt/PKB kinase, which has potent antiapoptotic and growth stimulatory effects. Therefore, PTEN can prohibit downstream signaling molecule activation and ultimately inhibit cellular proliferation, growth, and survival [15–18]. AFP binding to PTEN results in PTEN function deficiency, which can cause accumulation of PIP3 and the activation of AKT. Activation of AKT stimulates cell cycle progression and can lead to disorders of cell proliferation, apoptosis, migration, and adhesion, which are associated with cancer initiation, progression, and metastasis [15, 16].

Although we have recently provided some evidence to demonstrate that cytoplasmic AFP regulates the phosphatidylinositol 3-kinase (PI3K)/AKT pathway in human hepatocellular carcinoma cells through binding to the PTEN protein, but the reason its function is different to HSA is unknown, and how AFP make its contact to PTEN is also unclear [14].

To further decipher these molecular mechanisms, we employed colocalization, coimmunoprecipitation (co-IP), and molecular docking approaches to analyze AFP and HSA interactions with PTEN. The results may be used to explain our existing data and design new experiments for verifying their interactions to identify the targets and establish novel therapeutic strategies for treating human hepatocellular carcinoma.

## 2. Results and Discussion

### 2.1. Intracellular Localization of AFP and HSA, and Interactions with PTEN

**Figure 1:**
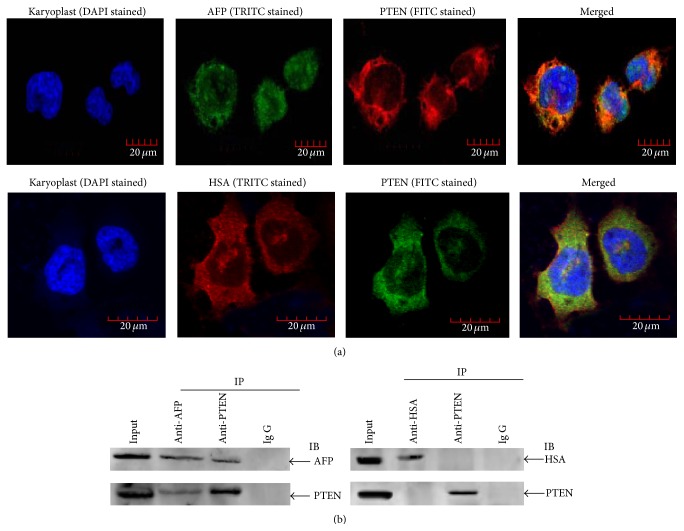
Colocalization, Coimmunoprecipitation analysis of the interaction of AFP and HSA with PTEN in PLC/PRF/5 cells. (a) Colocalization. Cells were cultured at 37°C in a humidified atmosphere of 5% CO_2_. Localization of AFP and HSA interaction with PTEN was viewed, and images were captured under laser confocal microscope. Nuclei were stained with DAPI (blue). PTEN were labeled with FITC (green) and AFP and HSA were labeled with FRITC (red), respectively. The image is representative of three independent experiments. (b) Coimmunoprecipitation (Co-IP) analysis of the interaction between AFP and PTEN. Lysates from PLC/PRF/5 cells were immunoprecipitated (IP) with antibodies against HSA, AFP, or PTEN and separated by SDS/PAGE gel electrophoresis. Coimmunoprecipitated complexes were transferred onto a nitrocellulose membrane, immunoblotted with anti-HSA, anti-AFP or anti-PTEN antibody, and scanned by the LAS3000 Chemiluminescence/Fluorescence Instrument (Fuji, Japan). Total protein immunoblotted with antibody against HSA, AFP, or PTEN is defined as input. Images are representatives of an experiment that was repeated three times.

The human liver cancer cells PLC/PRF/5 producing AFP were used to observe the colocalization of AFP and PTEN under a laser confocal microscope. The observation results showed the clear colocalization of AFP and PTEN in the cytoplasm of the cells but failed to show the colocalization of HSA and PTEN (Figure 1(a)). Results of Co-IP (coimmunoprecipitation) also demonstrated the binding of AFP and PTEN but did not show the binding of HSA and PTEN (Figure 1(b)). Recently, we have found that AFP stimulated expression of CXCR4 and Src through activation of the PI3K/AKT signal pathway in hepatoma cells [16]. These results imply that AFP might play a role in mediating the activity of PTEN in hepatoma cells.

### 2.2. Build 3D Structural Model of AFP

**Figure 2:**
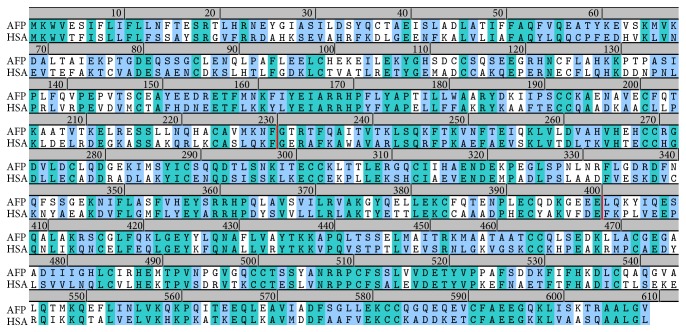
The sequence alignment of human alpha-protein (AFP) and human serum albumin (HSA). The identical amino acids are highlighted in green and the similar amino acids in light green. The proteins can be divided into three domains: the N-terminal region of 1–230 residues belongs to domain I, the middle region of 230–400 residues belongs to domain II, and in the C-terminal, the region of 400–609 residues belongs to domain III.

To further decipher the molecular mechanisms of AFP and HSA interaction with PTEN, we explored the combined* in silicon* methods (homology modeling, molecular dynamics simulations, and docking) for predicting their interactions with PTEN. A protein sequence search for alpha-fetoprotein (AFP) homologs in protein data bank (PDB) has revealed a good match in human serum albumin (HSA, PDB 4BKE). The protein sequences of alpha-fetoprotein (AFP) and human serum albumin (HSA) were aligned in pairwise and the alignment was shown in Figure 2. The sequence identity (the green residues) is 40% and the similarity (the blue residues) including the identity was about 60%. According to previous studies, this protein can be divided into three domains: The N-terminal region of 1–230 residues belongs to domain I, the middle region of 230–400 residues belongs to domain II, and in the C-terminal, the region of 400–609 residues belongs to domain III. The regions of domain II and III in AFP have higher sequence homology levels to HSA than domain I.

**Figure 3:**
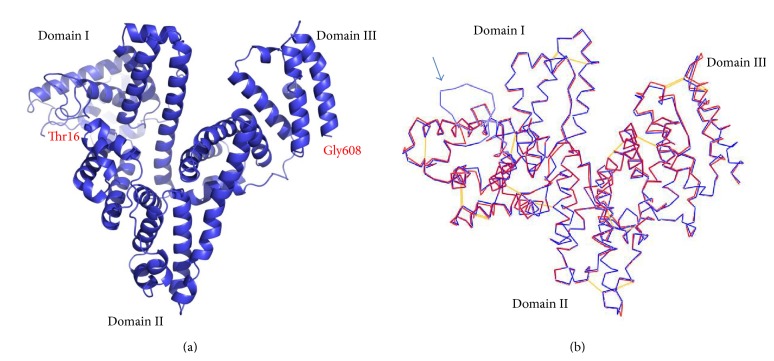
(a) AFP structure model. It exhibits a V-shaped structure, the domain I in the left side of V-shape (the N-terminal), domain III in the right side of V-shape (C-terminal), and domain II in the bottom of V-shape (middle region). (b) AFP structure model (blue line) overlaps with HSA structure (red line). The AFP structure model was generally well fitting with the structure of HSA, a notable difference is in the sequence position 25–50 region, and the AFP was obviously looped out (indicated by arrow).

Based on the HSA structure (PDB ID: 4BKE) [19], we conducted molecular modeling to predict the AFP structure using the program Modeler (version 9.0), which is based on protein sequence homology (sequence alignment) (Figure 2). After refinement and energy minimization, a 3-dimensional structural model of AFP was built (Figure 3(a)). This protein structural model of AFP is comprised of three domains and also exhibits a V-shaped structure as predicted by previous studies: the domain I at the left side of the V-shape (the N-terminal), domain III on the right side of the V-shape (C-terminal), and domain II in the bottom of the V-shape (middle region).

**Figure 4:**
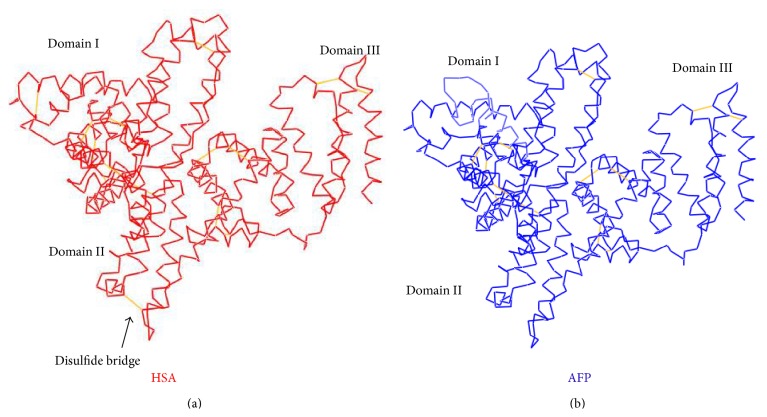
Comparison of HSA structure ((a) in red) and AFP model ((b) in blue). The disulfide bond is showed in the V-shaped region of domain II in HSA (a), but it is not present in AFP (b).

Comparing these two structures from the overlay (Figure 3(b)), the AFP structure model (blue line) generally fits well with HSA structure (red line); the most notable difference was in the amino acid sequence region from 25–50 of the AFP which was obviously looped out (Figure 3(b)). When we checked the sequence alignment, we found that this region aligned poorly since the sequences homology level was lower in domain I. The sequence differences in this region may cause the bioactive difference of AFP from HSA. Another notable difference was the disulfide bonding at the V-shape bottom in the domain II; HSA V-shaped had a disulfide bond (Cys340–Cys385) located between the two separated chains, (Figure 4(a)), but at the bottom of AFP, the V-shaped did not have a disulfide bond (Figure 4(b)). It is evident that this disulfide bond can stabilize the V-shaped domain II conformation in HSA. The V-shaped domain II of AFP may not be as stable as HSA. On the other hand, lacking this disulfide bond may confer more flexibility of movement to domain I and domain III which may provide a functional advantage to AFP. This could explain why the bioactivity of AFP is different from HSA.

### 2.3. AFP Docking to PTEN and MD Simulations of the Interfaces

**Figure 5:**
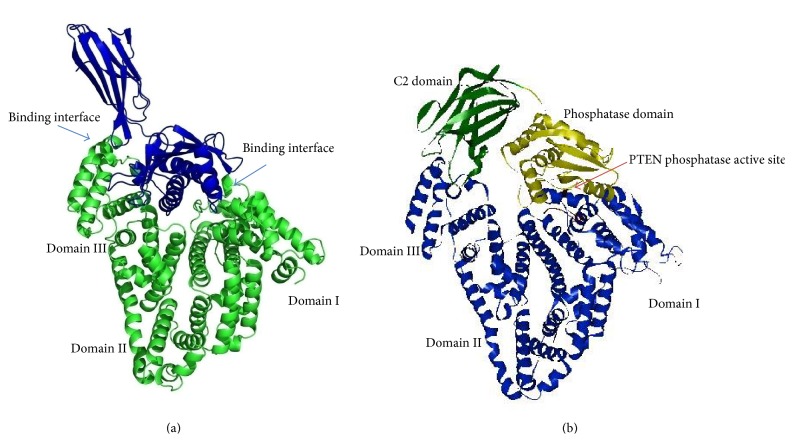
Overall structure of HSA/FcRn complex and AFP-PTEN complex model. (a) X-ray structure of HSA/FcRn complex and (b) structure model of AFP in complex with PTEN. The binding interface was similar to HSA/FcRn complex. PTEN C2 domain was shown in green and phosphatase domain was shown in yellow. AFP (in blue) domain I binds to PTEN phosphatase domain, and domain III binds to C2 domain. PTEN phosphatase active site was wrapped by AFP domain I.

Because AFP and HSA display high sequence homology and their 3-dimensional structures are similar, it is rationale for us to use the cocrystal structure of HSA/FcRn complex (PDB: 4K71) [20] as a reference for docking the AFP onto the PTEN. PTEN consists of two domains, the phosphatase domain and catalytic domain (C2), which are associated through an extensive interface. The C2 domain may serve the catalytic function domain on the membrane [21]. Using ZDOCK program, we have successfully docked AFP onto the PTEN. The architecture of AFP/PTEN complex (Figure 5(b)) was very similar to that of HSA/FcRn complex (Figure 5(a)). PTEN C2 domain was shown in green and phosphatase domain in yellow and AFP in blue. The AFP domain I contacted PTEN phosphatase domain, and domain III contacted PTEN C2 domain. The PTEN phosphatase active site appears to be wrapped by the AFP molecule. As a result of AFP binding, the PTEN would lose its function to prohibit the PI3K/AKT signaling molecule activation.

**Figure 6:**
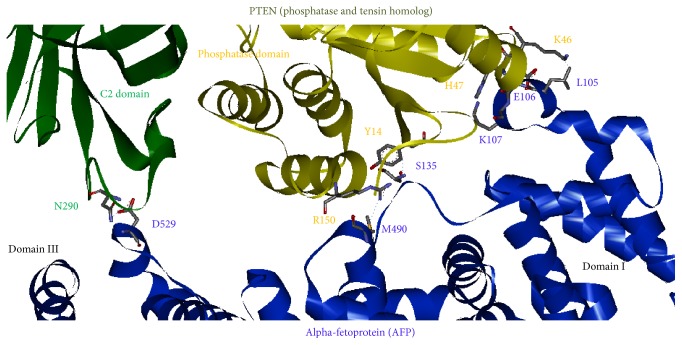
The binding interface of AFP and PTEN in the docking model, showing the individual residue contacts at the binding interface: AFP domain III residue D529 has a salt-bridge interaction with PTEN C2 domain residue N290; AFP domain I residue M490 has a hydrogen bond with PTEN phosphatase domain R150; residue S135 has a hydrogen bond with PTEN phosphatase domain residue Y14; domain I residue K107 has a hydrogen bond with H47. The L105 and E106 of AFP contacted with PTEN K46.

The contacting details of the individual residues involved in binding were shown in Figure 6. AFP domain III residue D529 has a salt-bridge interaction with PTEN C2 domain residue N290; the residue M490 has a hydrogen bond interaction with PTEN phosphatase domain R150. AFP domain I residues K107 and S135 form a hydrogen bond with PTEN phosphatase domain residues H47 and Y14, respectively. The L105 and E106 of AFP have close contact with PTEN residue K46.

As shown in Figure 2, the sequence alignment of AFP and HSA, we have tested the substitution of AFP binding site residues at the positions 490M/K and 105L/R with corresponding residues in HSA that had resulted in steric clashes with PTEN residues R150 and K46, respectively. Hence, the difference between these two residues might be contributed to HSA that could not bind to PTEN. Thus, our structural model of AFP-PTEN complex has explained these biochemical data that AFP could bind to PTEN and result in upregulating the P13K/AKT signal pathway.

## 3. Conclusions

We have used colocalization and coimmunoprecipitation approaches to demonstrate the relationship of AFP and PTEN and their function. We also showed, however, that HSA could not bind to PTEN. Molecular docking studies provided more details of AFP binding to PTEN through its domains I and III. The disulfide bond fixed the V-shaped region of domain II in HSA that was not present in AFP, which may be the explanation for the bioactivity differences in AFP and HSA. The binding site residues 490M and 105L may be critical for AFP binding to PTEN. Furthermore, our docking model could provide us more information for designing drug or vaccines in treating and preventing human hepatocellular carcinoma.

## 4. Materials and Methods

### 4.1. Cells

Human liver cancer cell line PLC/PRF/5, which is an AFP-producing hepatoma cell lines, was purchased from Department of Cell Biology, Peking University Health Science Center. The cells were maintained in Dulbecco's modified Eagle's medium (DMEM) supplemented with 10% fetal calf serum and cultured at 37°C in a humidified atmosphere containing 5% CO_2_.

### 4.2. Coimmunoprecipitation and SDS-PAGE/Western Blot Analysis

Coimmunoprecipitation (Co-IP) experiments were used for evaluation of the interaction of AFP and PTEN in PLC/PRF/5 cells as described previously [2]. Mouse anti-human serum albumin monoclonal antibody and rabbit anti-human PTEN polyclonal antibody were purchased from Santa Cruz Biotech Inc.

### 4.3. Colocalization of AFP and PTEN in Human Hepatoma Cells Was Observed by Laser Confocal Microscopy

Human hepatoma PLC/PRF/5 cells were fixed in ice-cold paraformaldehyde solution (4%) followed by soaking in 0.3% Triton-X 100. Mouse anti-AFP and anti-HSA antibody and rabbit anti-human PTEN antibody were added for 12 h. Secondary goat anti-mouse or anti-rabbit IgG antibodies conjugated with fluorescence isothiocyanate (FITC) or rhodamine (TRITC) (Jackson Immuno Res Lab, Inc., USA) were applied for 2 h followed by addition of 10 *μ*L DAPI (2-(4-amidinophenyl)-1H-indole-6-carboxamidine, 100 *μ*g/mL). Cells were viewed and captured with a Laser Confocal Microscopy (Leica TCS-NT SP2, Germany).

### 4.4. Sequence Alignment, Molecular Modeling, Docking, and Simulation

The sequence of AFP (accession number: EAX05681.1) was obtained from NCBI Genbank, and the structural files were obtained from protein data bank (PDB) as follows: HSA (PDB: 4BKE) [19], PTEN (PDB: 1D5R) [21], and HSA/FcRn complex (PDB: 4K71) [20]. The AFP modeling was conducted using program Modeler version 9.0, and docking was conducted using program ZDOCK. The CHARMm was used for all simulations [22, 23].
